# Diet Hypotheses in Light of the Microbiota Revolution: New Perspectives

**DOI:** 10.3390/nu9060537

**Published:** 2017-05-24

**Authors:** Tomasz P. Wypych, Benjamin J. Marsland

**Affiliations:** Faculty of Biology and Medicine, University of Lausanne, Service de Pneumologie, CHUV, Epalinges 1066, Switzerland; benjamin.marsland@chuv.ch

**Keywords:** Western diet, nutrients, allergy, microbiota

## Abstract

From an evolutionary standpoint, allergy has only recently emerged as a significant health problem. Various hypotheses were proposed to explain this, but they all indicated the importance of rapid lifestyle changes, which occurred in industrialized countries in the last few decades. In this review, we discuss evidence from epidemiological and experimental studies that indicate changes in dietary habits may have played an important role in this phenomenon. Based on the example of dietary fiber, we discuss molecular mechanisms behind this and point towards the importance of diet-induced changes in the microbiota. Finally, we reason that future studies unraveling mechanisms governing these changes, along with the development of better tools to manipulate microbiota composition in individuals will be crucial for the design of novel strategies to combat numerous inflammatory disorders, including atopic diseases.

## 1. Introduction

Allergy is one of the leading health problems in industrialized countries, affecting around 50 million people in the United States alone, and the number of atopic individuals continues to grow. The hallmark of this disorder is a strong Th2 response with upregulated levels of the interleukin-4 (IL-4), IL-5, and IL-13, which leads to enhanced immunoglobulin E (IgE) and IgG1 production, cell recruitment to the site of allergen exposure, and exaggerated immune responses leading to tissue damage.

From an evolutionary perspective, allergies have only recently appeared as a significant health problem. Therefore, researchers have long linked their emergence with rapid lifestyle changes, which occurred in the course of hominine evolution. The “hygiene hypothesis” and its derivatives (the “old friend” and the “biodiversity” hypotheses) pointed to the reduced exposure to environmental microorganisms and helminths in industrialized countries nowadays [1,2,3]. The “toxin hypothesis” underlined the presence of plant-derived toxins in contemporary foods and skin-care products [4]. Dietary habits are another example of these rapid lifestyle changes and hence, different forms of “diet hypotheses” have emerged [5,6,7]. The introduction of animal husbandry and agriculture in the Neolithic period slowly initiated these shifts. The Industrial Revolution and development of better tools for food processing further escalated them. As a result, new food items were introduced, such as refined grains, sugars, and vegetable oils, or manufactured salt. In addition to this, development of a mechanical reaper in 19th century allowed for increased harvest of grains, which coincided with the development of steam engine and railroads—prerequisites for grain and cattle transportation. This created a habit of feeding cattle with grain [8], leading to increased saturated fatty acids (SFA) content and increased ratio of n-6 to n-3 polyunsaturated fatty acids (PUFAs) in their meat [9,10].

The bloom of the aforementioned foods fundamentally influenced nutritional characteristics of industrialized regions, increasing glycemic load (potential of food to increase blood glucose and, in turn, insulin levels), and altering various parameters, including fatty acid composition (elevating levels of n-6 PUFAs while reducing that of n-3 PUFAs), macro- and micronutrient intake (decreasing protein and vitamin/mineral density content, respectively), sodium-potassium ratio (increasing Na^+^ while reducing K^+^ levels), and finally, the fiber content (leading to its severe reduction) [8]. As an outcome, food in the 20th century substantially differed from what our ancestors consumed. Since these changes occurred so rapidly on an evolutionary scale, our genome could not have adapted to them. This notion stands behind the hypotheses that contemporary diet may contribute to development of so-called “lifestyle diseases”.

Unfortunately, developed countries did very little in the last few decades to spread awareness of this phenomenon and counteract it. The ubiquitous presence of highly processed, high fat, and high sugar food and drinks has been a major driver in the development of a so-called “Western diet”, in which the trends mentioned above are amplified to their extremes. For example, consumption of refined sugars in the United States increased by 24.5% between 2000 and 1970 while that of refined vegetable oil increased by 170% between the 1990s and the 1940s [8].

In this review, we will look at the epidemiological and experimental evidence that these changes may predispose individuals to develop allergies and reason why current trends in dietary habits of Western civilization might constitute a health threat.

## 2. Nutrients and Epidemiology of Allergy

One of the earliest notions that nutrients might influence allergic diseases was introduced in the late 1980s when an association between sodium intake and asthma was reported [11,12]. These data were backed-up by several other groups studying asthma in both adults and children [13,14,15], and although their conclusions were not consistently supported [16,17,18], they pioneered the notion that diet may influence development of allergy. Soon after the “sodium hypothesis”, other diet hypotheses emerged. In 1990, Schwartz and Weiss analyzed data from the Second National Health and Nutrition Examination Survey, taking into account various antioxidants, including vitamin C. This study revealed a negative association between vitamin C intake and bronchitis/wheezing [13], a conclusion that was supported by some, but not all, subsequent studies [19,20,21,22]. However, trials to control asthma progression via dietary vitamin C supplementation brought disappointing results, questioning the significance of vitamin C in allergy prevention [23,24]. Similar discrepancies were found for other antioxidants, such as vitamin E, β-caroten, or selenium [19,22,25,26].

Soon after the antioxidant hypothesis, the link between increased intake of n-6 and reduced consumption of n-3 PUFAs with atopy and asthma was suggested [27,28,29]. However, as in the case of the antioxidant hypothesis, observational studies brought inconsistent results, with some of them supporting it [30,31,32,33], some disputing it [34,35,36], and some even indicating the opposite correlation [37,38]. Similar to the antioxidant hypothesis, interventional studies aiming to improve asthma severity via modification of fatty acid intake have been disappointing [5,6,7].

One factor which might play a significant role is whether the nutrient is assimilated via diet modification or supplement administration. Observational studies cited in this manuscript relied on food questionnaires and blood or urine analysis and did not provide information regarding this issue [13,15,16,18,19,21,26,30,31,33,34,35,37,38]. Its potential importance is exemplified in the interventional study by Troisi et al., who dissected the influence of nutrients rich in vitamin C and E from vitamin C or E supplementation [25]. Interestingly, diet-derived vitamin E was inversely associated with asthma, while in the case of supplement-derived vitamin E, a positive association was found. Comparatively, there was no significant influence of diet-derived vitamin C on asthma, while supplement-derived vitamin C correlated positively with this condition [25]. Comparison of the impact of diet and supplement-derived nutrients on allergic conditions will be important for future studies.

## 3. Milk and Epidemiology of Allergy

Milk is not solely a source of nutrients but contains various other components, such as immunoglobulin A, cytokines, bacterial metabolites, and, in the case of unpasteurized milk, live bacteria. Considering this, we are describing the relationship between milk consumption and allergy in a separate paragraph, starting with the intake of milk from breastfeeding mothers in infancy, followed by consumption of unpasteurized milk.

### 3.1. Breastfeeding

Breastfeeding is one of the few features linking dietary habits of people today with that of our evolutionary ancestors. The sole fact that it is so highly conserved among mammals highlights its important physiological role. For this reason, it has long been speculated that breastfeeding may protect against development of atopic diseases. One of the first studies to support this idea was published in 1936 when an inverse relation between breastfeeding and infantile eczema was reported [39]. Since then, a number of studies supported the protective role of breastfeeding against atopy in infancy [40,41,42,43,44]. Importantly, Saarinen and colleagues followed up on individuals for 17 years, showing that the protective effect of breastfeeding was maintained during childhood and adolescence [45]. Also, additional studies focusing on children reached similarconclusions [33,46,47,48].

However, not all studies confirmed this. In the study by Hide and Gruyer initiated in 1981, breast-fed children had the same (in the case of asthma) or even increased (in the case of eczema) risk of developing atopic disease [49]. Also, Wright and colleagues reported that beginning at the age of 6 years, breastfeeding was no longer associated with protection from recurrent wheeze and, in fact, carried an increased risk in the case of atopic children with asthmatic mothers [50]. No protection against asthma or even elevated risk for its occurrence was also reported by other studies [51,52,53]. The reasons for these discrepancies are not clear, although they might have been caused by variation in milk composition between individuals. Of note, infant formulas have greatly changed over past decades, which constitutes another variable to consider. However, many studies cited above were performed in different decades but reached similar conclusions while others were performed in the same decade but reached conflicting results. These examples suggest that decade-dependent differences in infant formula composition may not be a major confounding factor.

In order to clarify the inconsistencies between the studies, meta-analyses of published reports were undertaken. An analysis of 12 studies concerning bronchial asthma pointed towards an inverse association between breastfeeding and occurrence of this disease during childhood [54]. Also, an analysis of 18 studies regarding atopic dermatitis reached similar conclusions, but the effect was restricted to children with a family history of atopy [55]. Another meta-analysis of six studies underlined a protective role of breastfeeding in the development of allergic rhinitis [56]. Finally, the most comprehensive meta-analysis regarding asthma to date, taking into account 117 studies, found that breastfeeding was a factor reducing the risk of childhood asthma, with the strongest association in infants (0–2 years) [57].

Collectively, although there is still controversy in the field, most studies conclude that breastfeeding protects infants and children from developing allergic diseases [58,59]. The underlying mechanisms are not clear at present but may be associated with immunological components of breast milk (e.g., immunoglobulin A and its immune complexes, cytokines), antigens (e.g., allergens), prebiotics (e.g., human milk oligosaccharides), bacteria and bacterial metabolites, and/or others. Interestingly, many of these components (e.g., IgA [60], human milkoligosaccharides [61], human milk microbiota [62]) have the potential to influence the microbiota and the homeostasis of infant’s intestines, which may be of importance for the development of immune tolerance later in life. Detailed description of these putative mechanisms is beyond the scope of this review. Instead, the reader is referred to other recent publications in this field [61,62,63,64,65,66].

### 3.2. Unpasteurized Cow’s Milk

It has been well documented that growing up on a farm protects against development of allergies [67]. One of the proposed factors playing a role in this protection is consumption of raw milk. Indeed, a cross-sectional survey in rural areas of Austria, Germany, and Switzerland inversely associated raw milk consumption with asthma, hay fever, and allergic sensitization [68]. This conclusion has been supported by a cross-sectional multi-center study including almost 15,000 children from five European countries [69]. Also, similar results were obtained by others. For example, Wickens and colleagues found a protective effect of unpasteurized milk consumption against atopic eczema/dermatitis syndrome among farm children from New Zealand [70]. Perkin and Strachan found that consumption of unpasteurized milk protected against development of eczema and atopy in rural England [67]. Finally, the GABRIELA study reported an inverse association between raw milk consumption and asthma, atopy, and hay fever in children from rural areas of Austria, Germany, and Switzerland [71]. Taken together, there is strong evidence that consumption of raw milk early in life protects against development of allergies, perhaps even more convincing than the possible protective effect of breastfeeding. On the other hand, it must be emphasized that raw milk may contain certain human pathogens, such as *Salmonella* spp., *Campylobacter* spp., human pathogenic *Escherichia coli*, and *Listeria monocytogenes*. Therefore, its consumption carries risk for serious infectious diseases and milk processing (pasteurization or ultra heat treatments) effectively minimizes this risk. For this reason, raw milk consumption is not a general solution for allergy prevention. Instead, identification of the mechanisms behind its protective action will be essential for designing novel prophylactic and therapeutic strategies for allergic diseases. Similarly as in the case of breast milk, various mechanisms may play a role, including bovine immunoglobulins, cytokines and oligosaccharides [72], fatty acids [73], miRNA [74], antigens [71], milk microbiota [75], and others. Detailed description of these potential mechanisms is beyond the scope of this review and has been covered elsewhere [72,76].

## 4. Dietary Fiber and the Lung Function

Western diets are often rich in fat and processed foods, but low in fiber [8]. For this reason, a notion that fiber consumption may reduce symptoms of asthma has been hypothesized [77]. This has been based upon studies using mouse models (discussed in the next chapter) as well observational studies linking fiber intake with improved lung function [78,79,80,81]. Kan and colleagues were the first to observe a positive association between fiber intake and better lung function in chronic obstructive pulmonary disease (COPD). Statistically significant trends were found for total fiber intake, cereal fiber, and fruit fiber [78]. Similar conclusions were reached by Varasso et al., who found a negative association between total and cereal fiber intake and the risk of newly diagnosed COPD [79]. An inverse association between dietary fiber and impaired lung function was also found in the case of asthma. Berthon and colleagues compared dietary intake patterns between patients suffering from severe persistent asthma and healthy individuals. Interestingly, a positive association was found for fat (total and monounsaturated fatty acids) and sodium intake while an inverse correlation was observed for fiber and potassium intake [80]. Finally, Root et al. surveyed 15,567 American subjects for their dietary habits and correlated calculated macronutrient intake with their lung function. Total calories as well as saturated fatty acids were inversely associated with the pulmonary function [measured by the ratio of forced expiratory volume in 1 second to forced vital capacity (FEV1/FVC)], while for dietary polyunsaturated fatty acids, long-chain omega-3 fatty acids, dietary fiber, and animal protein, a positive correlation was found [81]. Given the above evidence, Halnes and co-workers hypothesized that high-fiber meal challenge may ameliorate symptoms in asthmatic patients. They recruited 29 individuals with stable asthma and divided them in two groups: 17 patients were challenged with a soluble fiber meal and 12 of them with a control meal. Interestingly, patients receiving a soluble fiber meal had decreased levels of several airway inflammation biomarkers 4 hours post-challenge, including exhaled nitric oxide, sputum total cell, neutrophil, lymphocyte, and macrophage counts as well as sputum IL-8 protein concentration. Intriguingly, these changes correlated with increased expression of GPR41 and GPR43 in the sputum of these patients, suggesting the mechanistic basis for these beneficial changes [77]. The possible importance of GRP41 and GPR43 in triggering anti-inflammatory mechanisms downstream of fiber intake will be discussed in detail in the next chapter.

In conclusion, various nutrients have been proposed in the past to influence risk for allergy development and many epidemiological studies have been launched to investigate their impact. Although “diet hypotheses” are still controversial, it seems that certain nutrients may indeed confer protection, especially in the case of consumption of raw milk. However, epidemiological studies regarding many other nutrients are scarce, including intake of dietary fiber, which has dramatically reduced over the last centuries. In the next section, we will discuss the potential role for dietary fiber intake in protection against diseases, including allergy, based on experimental data using mouse models.

## 5. Dietary Fiber, Short-Chain Fatty Acids, and Susceptibility to Diseases: Lessons Learned from Animal Studies

Anti-inflammatory properties of butyrate, a short-chain fatty acid produced by fermentation of soluble fibers by commensal bacteria in the gut, have long been recognized. Although initially proposed to exert its function through restoration of energy metabolism in colonocytes [82], later research rather supported its immunomodulatory properties, based on experiments in vitro [83,84,85,86]. Apart from the butyrate, in vitro anti-inflammatory properties of other short-chain fatty acids, such as propionate and acetate, have also been proposed [87].

An important step towards unraveling the mechanisms behind short-chain fatty acids (SCFA) action came with identification of G-protein-coupled receptors GPR41 and GPR43 as their extracellular receptors [88,89]. The physiological importance of this finding was first highlighted in a study showing that the protective role of acetate in a mouse model of colitis was dependent on GPR43 [90], although this did not appear to be the case in a mouse model of allergic inflammation, where the effects of acetate did not require GPR43 [91]. Similar discrepancy was observed in the case of propionate. While exerting its anti-inflammatory properties via GPR43 in a mouse model ofcolitis [92], in a mouse model of allergic airway inflammation it was GPR41 but not GPR43 that played a major role [93]. The reasons for these differences are not clear. Finally, GPR109a has been reported as a low-affinity receptor for one of the short chain fatty acids, butyrate [94], and the physiological importance of this finding has been demonstrated in a mouse model of colitis [95,96] and food allergy [97].

Regardless of the surface receptor responsible for the initial recognition of short-chain fatty acids, many studies have focused on downstream mechanisms mediating their anti-inflammatory properties. For example, propionate and butyrate were shown to induce differentiation of regulatory T cells in vitro and in vivo and this process coincided with increased histone H3 acetylation inTregs [92], or more specifically, of *Foxp3* regulatory elements [98,99]. Smith and colleagues proposed that propionate acts directly via GPR43 on colonic Tregs to induce these effects [92]. However, Arpaia et al. pointed out that in the case of butyrate, in addition to imprinting epigenetic effects directly on Tregs, it may also endow dendritic cells with superior capacity to drive differentiation of this subset [99]. Similar conclusions were drawn by Singh and colleagues who demonstrated that colonic dendritic cells (DCs) and macrophages from GPR109a-deficient mice were defective in inducing Treg cell differentiation in vitro [95]. Interestingly, in the mouse model of airway allergic inflammation, anti-inflammatory properties of propionate were not linked to Treg cells but rather to DC function, since propionate treatment in vivo did not affect Treg cell numbers but impaired the ability of dendritic cells to drive Th2 responses [93]. Finally, Macia et al. implicated inflammasome activation as a mechanism through which short chain fatty acids confer protection in a dextran sulfate sodium (DSS)-induced mouse model of colitis [96].

The mechanisms behind anti-inflammatory properties of acetate are also controversial. Furusawa et al. [98] and Arpaia et al. [99] suggested that acetate, unlike propionate and butyrate, lacks HDAC inhibitory properties and fails to induce Treg differentiation in vitro and in vivo. In the study by Fukuda et al., the authors suggest that the protective effects of acetate in their model of enteropathogenic infection may rely on its ability to induce anti-apoptotic and anti-inflammatory gene expression in colonic epithelial cells as well as on its capacity to increase transepithelial electrical resistance [100]. However, Thorburn and colleagues reached different conclusions based on their model of allergic airway inflammation. They observed increased acetylation levels of histones at the *Foxp3* promoter, elevated numbers of Treg cells and their enhanced suppressive activity upon feeding mice with acetate in the drinking water. Importantly, they concluded that the protective effect of acetate in their model of allergic inflammation was dependent on this subset of cells, as Treg depletion abrogated its beneficial role [91].

Since short-chain fatty acids are mostly products of bacterial fermentation of nutrients, the question regarding the interplay between SCFA production, diet, and microbiota composition has been raised. In a study from our group, some light onto these complex interactions was shed. Mice fed on a high fiber diet had increased ratio of *Bacteroidetes/Firmicutes* abundance in the gut and lungs and this coincided with increased cecal and serum levels of SCFA [93]. A similar observation was reported in the study by Thorburn and colleagues [91]. Of note, *Bacteroidetes* are known to be efficient at fermenting fiber into SCFAs, supporting a causative relationship between increased SCFA and the increase of *Bacteroidetes*. Nevertheless, since SCFA may also be produced by other phyla, the importance of *Bacteroidetes* increase in these models should be investigated further. Overall, it is important to note that high fiber diet protected mice against allergic airway inflammation, underlining that protective effects of SCFA are not restricted to the gut, but can influence other peripheral tissues [91,93].

The importance of high fiber diet-induced microbiota changes has also been implicated in a mouse model of colitis [96]. The authors linked the protective role of high fiber diet in this model with inflammasome activation. Interestingly, re-colonization of germ-free mice with microbiota from mice fed on a high fiber diet resulted in increased levels of IL-18 secretion and caspase-1 activity in comparison to the control group [96]. Further insights into the diet-microbiota-SCFA axis were gained by Tan and colleagues [97]. First, they noted that high fiber diet, which protected mice against peanut allergy, changed intestinal microbiota composition and increased levels of SCFA. In order to dissect the impact of these two factors, they re-colonized germ-free mice with fecal matter from mice fed on low-fiber or high fiber diets and showed that the latter were protected against peanut allergy despite having similar levels of SCFA. This indicated that the protective effect of fiber feeding in this model was not due to these metabolites. However, SCFA supplementation was also able to confer similar protection. Therefore, the authors propose that two mechanisms play a role upon feeding mice with a high fiber diet. Importantly, the effects of this diet relied on epithelial GPR43 and immune cell GPR109a, since feeding GPR43 or GPR109a-deficient mice with high fiber diet no longer protected mice against peanut allergy [97].

## 6. Dietary Fats and Susceptibility to Diseases

As previously mentioned, Western diet contains elevated levels of dietary fats [8]. For this reason, it has long been hypothesized that higher fat intake might be implicated in elevated risk for disease occurrence, including allergy. High fat diet-induced obesity could have a significant contribution to this. Indeed, a positive association between obesity and allergy is well documented in epidemiological studies [101,102,103,104] as well as in animal models of allergy [105,106,107,108]. Description of the current knowledge regarding this issue is beyond the scope of this review. Instead, the reader is referred to several recent reviews in this field [109,110,111,112]. However, it could be hypothesized that high fat diet enhances susceptibility to allergy independently of obesity. Increased free fatty acid release itself could be immunomodulatory and influence disease susceptibility. The effects of high fat meals independently of obesity have been shown in asthmatic patients. In the study by Wood et al., non-obese asthmatic patients receiving a high-fat meal had increased levels of TLR4 mRNA and neutrophils in their sputum cells in comparison to subjects receiving a low-fat meal [113]. In an animal model of allergy, although pups born from mothers fed on a high fat diet did not have increased body weight or blood glucose levels, they displayed a more severe anaphylaxis score after oral sensitization to peanut protein [114]. This study underlined transgenerational effects of high fat diet independently of major confounders, such as obesity or diabetes; however, the exact components of a high fat diet which could imprint these changes were not defined. Saturated fatty acids, which are major components of a high fat diet, could be involved, as their pro-inflammatory potential is well established [115]. Polyunsaturated omega-6 fatty acids could also contribute to this, as they have been described to enhance allergic responses in mouse models of asthma [116,117,118]. Finally, monounsaturated fatty acids are also candidates, as they constitute a major component of high fat diets, although their immunomodulatory potential in the context of allergy remains unexplored.

Overall, high fat diet may influence susceptibility to allergy through obesity or directly through nutritional composition. Regarding the latter, dietary fatty acids contained within the diet have the potential to induce pro-inflammatory responses. Decreased content of dietary fiber in high fat diets may at the same time lead to downregulation of anti-inflammatory pathways, further escalating the imbalance between pro- and anti-inflammatory responses. Further research is needed to establish the potential of dietary components of high fat diets to influence allergic responses and decipher the molecular mechanisms they trigger.

## 7. Conclusions and Perspectives

**Figure 1:**
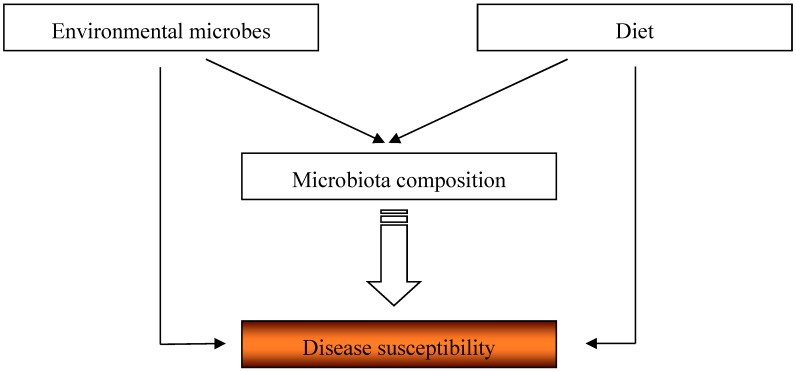
The cross-talk between environmental microorganisms, diet and microbiota composition and its impact of disease susceptibility.

Lifestyle changes, which most rapidly occurred in the last century, have been proposed to increase susceptibility to allergies. The hygiene hypothesis suggested the role of decreased contact with environmental microbes and helminths in this phenomenon, while diet hypotheses pointed towards the importance of changes in dietary habits. The microbiota seems to be a common component of these two views, as it is shaped by various external factors, including environmental microorganisms and diet (Figure 1). Given the vast impact the microbiota exerts on immune responses and susceptibility to diseases, it is crucial to integrate both views and understand how environmental cues influence microbiota composition. Unraveling this may lead to clearer distinctions between pathogenic and beneficial species and indicate ways to manipulate them. This holds promise for the development of novel therapeutic approaches targeting the microbiota for prevention and treatment of inflammatory disorders.
